# Finite Element Evaluation of the Electric Field Distribution in a Non-Homogeneous Environment

**DOI:** 10.3390/bioengineering10091062

**Published:** 2023-09-08

**Authors:** Elisabetta Sieni, Monica Dettin, Annj Zamuner, Maria Teresa Conconi, Bianca Bazzolo, Cristian Balducci, Paolo Di Barba, Michele Forzan, Patrizia Lamberti, Maria Evelina Mognaschi

**Affiliations:** 1 Department of Theoretical and Applied Sciences, University of Insubria, Via Dunant 3, 21100 Varese, Italy; 2 Italian Interuniversity Center ICEMB (Interaction between Electromagnetic Fields and Biosystems), DIET University of Genoa, 16145 Genoa, Italy; plamberti@unisa.it (P.L.); eve.mognaschi@unipv.it (M.E.M.); 3 Department of Industrial Engineering, University of Padua, Via Marzolo 9, 35131 Padua, Italy; monica.dettin@unipd.it (M.D.); cristian.balducci@studenti.unipd.it (C.B.); michele.forzan@unipd.it (M.F.); 4 Department of Civil Environmental and Architectural Engineering, University of Padua, Via Marzolo 9, 35131 Padua, Italy; annj.zamuner@unipd.it; 5 Department of Pharmaceutical and Pharmacological Sciences, University of Padua, 35131 Padua, Italy; mariateresa.conconi@unipd.it (M.T.C.); bianca.bazzolo@studenti.unipd.it (B.B.); 6 Department of Electrical, Computer and Biomedical Engineering, Pavia University, Via Ferrata 5, 21100 Pavia, Italy; paolo.dibarba@unipv.it; 7 Department of Information and Electrical Engineering and Applied Mathematics, University of Salerno, Via Giovanni Paolo II 132, 84084 Fisciano, Italy

**Keywords:** finite element analysis, conductivity inhomogeneity, electroporation, 3D scaffold

## Abstract

Finite element analysis is used in this study to investigate the effect of media inhomogeneity on the electric field distribution in a sample composed of cells and their extracellular matrix. The sample is supposed to be subjected to very high pulsed electric field. Numerically computed electric field distribution and transmembrane potential at the cell membrane in electroporation conditions are considered in order to study cell behavior at different degrees of inhomogeneity. The different inhomogeneity grade is locally obtained using a representative model of fixed volume with cell–cell distance varying in the range of 1–283 um. The conductivity of the extracellular medium was varied between plain collagen and a gel-like myxoid matrix through combinations of the two, i.e., collagen and myxoid. An increase in the transmembrane potential was shown in the case of higher aggregate. The results obtained in this study show the effect of the presence of the cell aggregates and collagen on the transmembrane potential. In particular, by increasing the cell aggregation in the two cases, the transmembrane potential increased. Finally, the simulation results were compared to experimental data obtained by culturing HCC1954 cells in a hyaluronic acid-based scaffold. The experimental validation confirmed the behavior of the transmembrane potential in presence of the collagen: an increase in electroporation at a lower electric field intensity was found for the cells cultured in the scaffolds where there is the formation of collagen areas.

## 1. Introduction

Electrochemotherapy (ECT) is a well-established therapy used in Europe and other countries to treat some types of tumors like melanoma or breast cancer recurrence after mastectomy, sarcomas, liver carcinoma, head and neck tumors, or others [1,2,3,4,5]. ECT uses a pulsed electric field in the presence of a chemotherapy drug to modify the structure of the membrane in a reversible condition to improve the drug uptake by the cells [6]. Elec troporation (EP) of the cell membrane occurs when the transmembrane potential (i.e., the difference in the electric field potential across the cell membrane) overcomes a prescribed threshold; thus, it is related to the local distribution of the electric field. The effect of the medium’s electrical characteristics have been already studied, considering cells suspended in buffer with different compositions and different electrical properties [7,8,9,10]. Electroporation of cells in suspension and in adherent cultures is usually carried out using an electroporation buffer, i.e., a phosphate buffer solution, which is a solution with a lower conductivity than the culture medium [11]. The typical EP buffer composed of phosphate and sucrose shows a conductivity of 0.2 S/m [10,12]. In the literature, some studies were performed using cells in suspension [9,10,13], and the analysis of several media showed that low conductivity media led to an improvement in EP efficiency and conductivity during EP [10,12].

Another issue in EP is the role of the extracellular matrix (ECM): membrane permeabilization is related to the local electric field intensity that modifies the transmembrane potential, and it is a function of tissue characteristics [14,15]. In particular, the electrical properties of tumors depend on tissue type (e.g., fat, muscle, etc.) and stroma [16,17,18,19,20] that contains a complex network of fibrous proteins embedded in hydrated glycosaminoglycans [21,22]. The stroma complexity is not mimicked by the two-dimensional (2D) in vitro models (cell suspensions or adherent cultures). Consequently, 2D models are unable to reproduce the electrical properties of tumor microenvironments. On the other side, the 3D models, like spheroids, mimic the cell–cell interaction [23,24] but not the effect of the extracellular environment. In a previous work, we proposed a 3D in vitro model in which breast cancer cells were cultured on hydrogels composed of hyaluronic acid (HA) enriched with self-assembly peptides carrying the IKVAV adhesion motif [25,26]. In these cultures, the cells produced abundant ECM, as demonstrated by histological staining [26]. Moreover, the same work showed a higher EP efficiency when a lower electric field strength was used with respect to the value suggested by the ESOPE protocol [4,6]. Thus, we have hypothesized that the local inhomogeneity in electrical properties due to ECM might affect the electric field distribution. This statement is supported here by computation using finite element analysis (FEA) as described by other authors [27,28]. Cell–cell distance and stroma conductivity are considered variables of our system.

**Figure 1:**
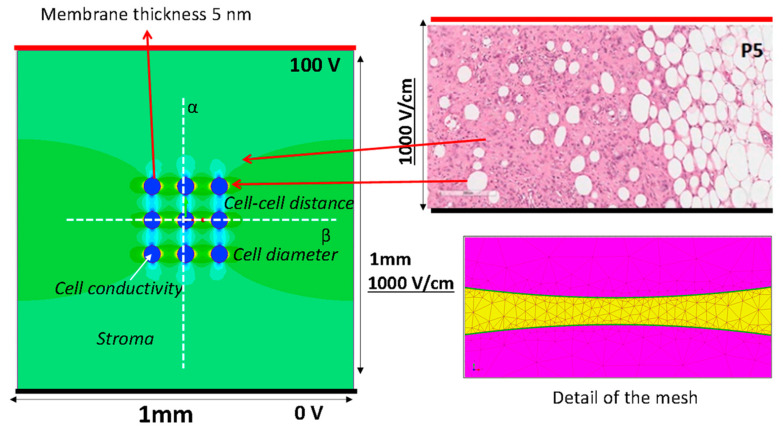
The geometry of the model used in the FEA (**left side**) mimics a histological hematoxylin/eosin staining of a sarcoma slice (**top right**), and an example of data analysis (i.e., color map showing electric field distribution). Representation of the lines used to model the electrodes (thick black lines supplied at 0 V and thick red line at 100 V). Electric field potential is evaluated along the white dotted lines reported in the model. Detail of the mesh used to compute the electric field (**bottom right**).

The idea of the FEA model to evaluate the effect of cell distance in the presence of a material inhomogeneity starts from histological hematoxylin/eosin staining of a sarcoma slice (image in the top right corner of Figure 1) [19]: in the represented area, the histological sample shows cells well separated with a fibrous stroma (collagen-based) and an area where cells were close to each other. Authors studied how the effect of cell aggregation and stroma conductivity affect EP, evaluating the transmembrane potential that was widely studied considering different parameters [29,30,31]. In fact, in the literature, some authors modeled the behavior of the single cell [30,32,33] as well as investigating the transmembrane potential in multicellular structures [34]. Moreover, the influence of electrical conductivity on electropermeabilization while also considering the membrane’s pore formation was studied in [7,9,35].

The ECM locally modifies the conductivity of the treated volume, so this study is established to understand what occurs at the electric field strength distribution in EP conditions in the presence of inhomogeneity, i.e., in a tissue where cells could be both immersed in stroma with different electrical characteristics (e.g., fibrous with a conductivity close to that of collagen [10,36] at 0.2 S/m or myxoid with a conductivity close to a liquid medium, e.g., 1.3 S/m) or grouped closely together in a collagen shell. Moreover, the case in which cells are isolated (lower cell density) but surrounded by a collagen shell is studied, too. The focus of this work is the evaluation of what occurs to the transmembrane potential when electroporation pulses are applied to the sample in some relevant cases that include one or more of the described conditions. The first pulse condition is considered in the modification of the transmembrane potential before pore formation. The simulated cases are validated by an experimental case.

## 2. Materials and Methods

### 2.1. Electric Field Distribution Using FEA

In this paragraph, the finite element model used for the numerical evaluation of the electric field is described. The model considers the instant before pore formation. A simple 2D numerical model, a square with 1 mm × 1 mm sides that includes 9 cells with spherical shape was simulated by Finite Element Analysis (FEA) to estimates the electric field distribution and the transmembrane potential. The 1 × 1 mm slice was supplied with a voltage difference applied to 2 parallel edges, the electrodes, as reported in Figure 1. To obtain an electric field of 1000 V/cm, the top side is energized at 100 V and the bottom side at 0 V, i.e., the thick red and black lines in Figure 1, respectively. The 9 blue circles represent the cells, with the border made of non-conductive materials, the lipidic bilayer (*σ* = 5.3 10^−6^ S/m), which encapsulates the cell interior made of conductive material with conductivity *σ_C_*. The cell diameter considered in the FEA is 10 μm or 50 μm (membrane thickness of 10 nm), and the cell–cell distance varied between 1 to 283 μm corresponding to a local density of cells between 8000 and 9 cell/mm^2^, depending on the cell diameter. The studied cases are summarized in Table 1. The material between the electrodes and outside the cells, the stroma, is a homogeneous conductive material with fixed electrical conductivity, *σ_S_*. Typical values used in the simulation are reported in Table 1 [37,38].

Electric field strength and the electric potential due to a voltage applied between the electrodes is computed using FEA, solving Maxwell’s equations in static conditions. The color map on the left side of Figure 1 is an example of the computed electric field strength. In particular, a conduction problem is analyzed, as proposed by other research groups [28,39]. The electric field intensity E(P) is evaluated at each spatial point P = (*x*,*y*) by means of a commercial finite element simulator (Simcenter Magnet, https://plm.sw.siemens.com/it-IT/simcenter, Germany, accessed on 4 September 2023) solving a static conduction problem in direct current on electric scalar potential, i.e., V linked to E(P) as E(P) = −∇*V*, and imposing a constant voltage (i.e., a Dirichlet boundary condition) on the lines that represent the electrodes (0 and 100 V in opposite lines—Figure 1) [28]. The Laplace equation in the scalar potential *V*, is solved [40,41]:

(1) ∇⋅σ∇V=0

where *σ* is the electrical conductivity of the material. Moreover, a tangent condition of electric field lines is assumed on the remaining two sides (i.e., a Neumann boundary condition for which the first derivative of the scalar potential *V* is null with respect to the normal direction, *n*, on the boundary line):

(2) $$\frac{\partial V}{\partial n}=0\;\mathrm{on external boundary}$$

**Figure 2:**
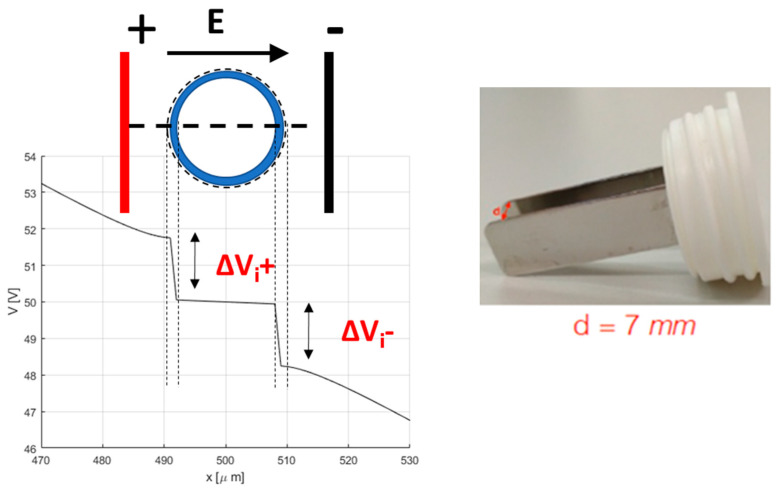
Schema for the determination of the transmembrane potential, and the electrode used in electroporation.

From the FEA, the color map of the electric field strength is extracted (an example is shown in Figure 1). The electric field strength is analyzed along the lines α and β in Figure 1, whereas the electric potential is only analyzed along the line α. In Figure 2, an example of the scalar potential behavior along the α-line, that is, the *x* coordinate in the schematic here adopted, is reported. The transmembrane potential *i*-th, Δ*V_i_*, is evaluated by considering the computed electric potential in two points close to the membrane, one outside and one inside the cell region (i.e., the points detected by the dashed lines in Figure 2) in the modeled non-homogenous domain that generally differs from the one in the case of a single cell immersed in an uniform distributed field given by [42,43,44,45]:

(3) $$\Delta Vi=fR_{1}E_{ext}cos\alpha_{p}$$

where *R*_1_ is the cell radius; *E_ext_* is the applied electric field; *f* is the shape factor that for round cells is 1.5; and *α_p_* is the angle between the electric field direction and a point on the cell surface. Different cases are investigated by varying the cell-to-cell distance, the cell diameter, the stroma conductivity *σ_S_*, and the cell interior conductivity *σ_C_*, as shown in Table 1. Column E in Table 1 shows the example of a model where the stroma conductivity is made of two materials: one half of the model is characterized by a value of conductivity different from the other half.

### 2.2. Scaffold Preparation and Cell Culture

#### 2.2.1. Materials for Scaffold Preparation

Hyaluronic acid (MW = 100–1250 kDa) was obtained from Contipro Biotech s.r.o. (Dolni Dobrouc, Czech Republic). 1-Ethyl-3-(3-dimethylaminopropyl)carbodiimide (EDC) and triethoxysilane (TES) were obtained from Sigma Aldrich (Steinheim, Germany), and ethanol was procured from VWR Chemicals Prolab (Fontenay-sous-Bois, France). The Rink Amide MBHA resin and the 9-fluorenylmethoxycarbonyl (Fmoc) protected amino acids were purchased from Novabiochem (Merck KGaA, Darmstadt, Germany). The coupling reagents 2-(1H-benzotriazole-1-yl)-1,1,3,3-tetramethyluronium hexafluorophosphate (HBTU) and 1-hydroxybenzotriazole (HOBt) were obtained from Advanced Biotech (Seveso, MI, Italy). N,N-diisopropylethylamine (DIEA) and piperidine were purchased from Biosolve (Leenderweg, Valkenswaard, The Netherlands). N,N-dimethylformamide (DMF), trifluoroacetic acid (TFA), N-methyl-2-pyrrolidone (NMP), and dichloromethane (DCM) were procured from Biosolve (Leenderweg, Valkenswaard, The Netherlands). Acetonitrile and TFA were obtained from Sigma-Aldrich, Saint Louis, MO, USA.

#### 2.2.2. Synthesis of an SAP Functionalized with Laminin Adhesion Sequence

The self-assembling peptide (SAP) used is an analogue of EAK 16 module II [46,47,48]. EAK 16 module II is a 16-mer peptide in which pairs of negatively (E, glutamic acid) charged residues are alternated with pairs of positively (K, lysine) charged residues, and each polar amino acid is separated from the following polar residue by a hydrophobic amino acid (A, Ala, alanine) [49]. The analogue of EAK 16, used in this study, presents the substitution Ala→Abu (Abu = α-aminobutyric acid) and the addition of a Laminin sequence IKVAV (Ile-Lys-Val-Ala-Val) at its C-terminus. The peptide was synthesized by Fmoc chemistry using Rink Amide MBHA resin (0.7 mmol/g; scale 0.125 mmol) and the synthesizer Syro I (Multisynthec, Witten, Germany). The first three amino acids and the last sixteen amino acids are introduced through double couplings [50]. At the end of the synthesis, the Fmoc is removed, the resin is washed with DCM and dried for 1 h under vacuum [50]. The peptide is cleaved from the solid support with contemporary side-chain deprotection using the following mixture: 0.125 mL MilliQ water, 0.125 mL TES, and 4.750 mL TFA over 90 min, under magnetic stirring. The resin is filtered, and the reaction mixture is concentrated. The crude peptide is precipitated with cold diethyl ether. The peptide is purified using reverse-phase high-performance liquid chromatography, and its identity is ascertained using MALDI-TOF (matrix-assisted laser desorption ionization–time of flight) mass spectrometry (theoretical value = 2239 Da; experimental value = 2236 Da).

#### 2.2.3. Preparation of the 3D Scaffold

An SAP (4.2 mg, 0.12% *w*/*v*) is dissolved in 3.5 mL of MilliQ water under stirring. Hyaluronic acid (108 mg, 3% *w*/*v*) is slowly added to the solution. The dense solution is divided and weighed into the 5 wells of a chamber slide, frozen in liquid nitrogen, and lyophilized. The scaffolds (dimension: 8 × 10 × 5 mm) are cross-linked through a reaction with 60 mM EDC in 95% ethanol for 24 h. The scaffolds are washed both with ethanol and then MilliQ water (three times each) in an ultrasound bath for 1 min, and another 2 min without sonicating. Finally, the scaffolds are frozen at −20 °C and lyophilized. Similarly, the same protocol without the peptide produces control matrices of cross-linked hyaluronic acid only (HA).

#### 2.2.4. Cell Cultures

HCC1954 cells (derived from human ductal carcinoma cells, primary tumor), purchased from ATCC (Manassas, VA, USA) (https://www.atcc.org, accessed on 1 September 2023), are cultured in RPMI medium supplemented with 1% penicillin/streptomycin, 1% L-glutamine, and 10% FBS at 37 °C in a humified atmosphere with 5% CO_2_ (SteriCult CO_2_ incubator, Thermo Electron Corporation, Thermo Fisher Scientific, Cincinnati, OH, USA). Cells (1.8 × 10^5^) are seeded on hydrated scaffolds previously put into each well of an 8-well cell chamber slide. 

#### 2.2.5. Electroporation Procedure

EP is performed by applying a sequence of 8 voltage pulses, 100 µs long at 5 kHz, at different amplitudes to each well through a plate electrode. The electrode is formed by two 3 cm-long, 1 cm-large stainless-steel plates, with a gap of 7 mm (Figure 2). The voltage pulses are applied using a pulse generator EPS-01 (Igea, Carpi (MO), Italy). The electrode is immersed in the cell culture in medium. The applied voltages lead to electric fields ranging between 0 and 1000 V/cm (0, 400, 600, 800, and 1000 V/cm). These experiments are carried out in cells cultured in 2D in RPMI medium and in scaffolds, both HA-only and HA-EAbuK-IKVAV, to evaluate the influence of the extracellular matrix in the electric field intensity threshold to electroporate cell membranes. 

#### 2.2.6. Fluorescent Staining after Electroporation

The occurrence of EP is evaluated by adding 15 μL of propidium iodide, PI, (Sigma Aldrich, Saint Louis, MO, USA) solution (1 mg/1 mL in deionized water) immediately before the delivery of the voltage pulses. Then, EP 5 μL of Hoechst 33342, HOE, (ThermoFisher, Waltham, MA, USA), solution (1 mg/1 mL in PBS) is added. In particular, PI selectively stains in red the nucleus of electroporated cells [13,51], whereas HOE stains all the cells in the culture [26,51].

The 2D and 3D cultures are observed 20 min after staining with an inverted microscope Leica DI4000 (objective 20 × 0.35 DRY, camera DFC300FXR2-078921405) (Leica Microsystems Srl, Wetzlar, Germany): PI (excitation 538 nm, emission around 619 nm) and HOE (excitation 352 nm, emission around 455 nm) are visualized. For each culture, some blue and corresponding red images were recorded. Both blue and red fluorescence images are superposed through the software LAS AF Lite (Leica, https://www.leicabiosystems.com, Deer Park, IL, USA, accessed on 1 September 2023). 

## 3. Results

### 3.1. Numerical Analysis of Relevant Cases

**Figure 3:**
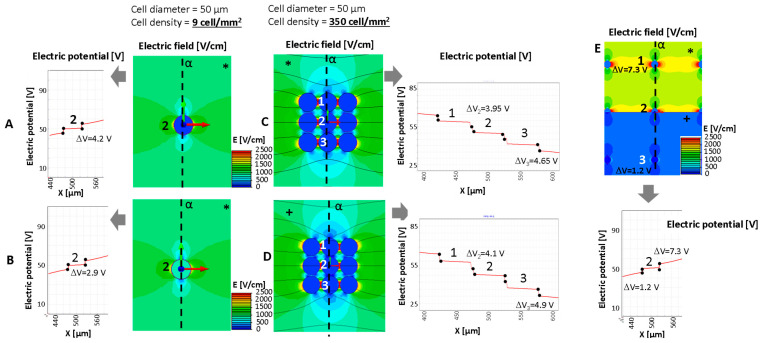
Electric potential for the evaluation of transmembrane potential and electric field distribution around a cell (**A**,**B**,**E**) or groups of cells (**C**,**D**) in some relevant cases (cell diameter 50 um): analysis of a single cell in fibrous stroma with low cell density and cell conductivity for cases described in Table 2. (**E**) Behavior of the electric potential, transmembrane potential, and electric field distribution in presence of stroma inhomogeneity. * fibrous stroma with *σ_C_* = 0.2 S/m. +: myxoid stroma with *σ_C_* = 1.3 S/m. The number 1–3 represents the cells for which the transmembrane potential is evaluated.

**Figure 4:**
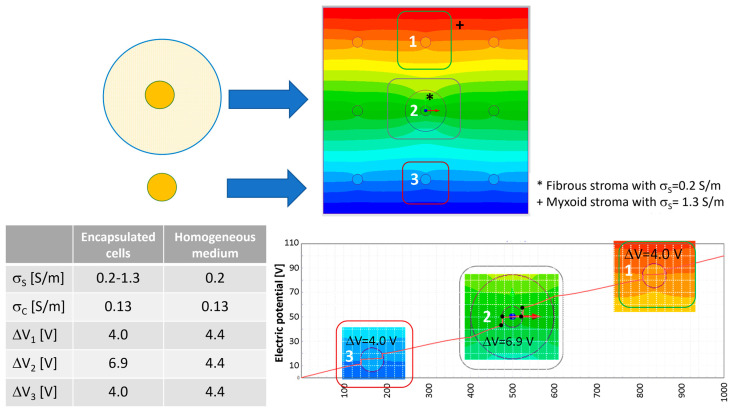
Electric potential map in the nine-cell group with a local cell density of nine cells/mm^2^ (cell–cell distance 283 μm, cell diameter 50 μm) and transmembrane potential for the cell surrounded by the fibrous shell without shell with a diameter of 200 μm. The number 1–3 represents the cells for which the transmembrane potential is evaluated.

Figure 3 and Figure 4 show the computation results in some relevant cases, considering nine cells with a different intercell distance in the simulated domain (1 × 1 mm) and a cell diameter fixed to 50 μm. The domain is energized by a couple of plate electrodes supplied with a voltage difference of 100 V able to generate a constant electric field of 1000 V/cm in homogeneous medium conditions. The reported results show that the electric field distribution and the consequent transmembrane voltage are influenced by the intracell material conductivity (named the stroma), *σ_S_*, the cell interior conductivity, *σ_C_*, and in some cases the cell distance and non-homogeneous stroma arrangement. From the color map of the electric field strength, it can be observed, as is well known, e.g., in [30,52], that the cells are able to locally modify the electric field strength distribution even if the stroma conductivity is homogeneous in all the domains (Figure 3A–D). The cells shown in Figure 3 have a cell diameter of 50 μm and a density of 9 cell/mm^2^ with a cell–cell distance of 283 μm for the cases in panels A, B, and E and a density of 350 cell/mm^2^ with a cell–cell distance of 3.5 μm for the cases in panels C and D.

Figure 3 compares, in terms of electric field distribution (color map), electric potential along α (x-axis in the reported behavior) and transmembrane potential, in the cases of well-separated cells and aggregated cells. The focus is on the cell labeled ‘2’ (which is in the center of the modeled group of cells) and neighbor cells labeled ‘1’ and ‘3’. Cases A and B in Figure 3 (well-separated cells) represent a cell and a cell with a lipid component (e.g., from a liposarcoma or similar), respectively. Both cases consider the same stroma. This example shows a decrease in the transmembrane potential, Δ*V*, in the case where the cell is filled with a lipid component (Figure 3B). Its value passes from 4.2 V to 2.9 V when the cell with an internal lipid is considered. On the other hand, panels C and D show that the cell proximity (e.g., as can occur in a spheroid) modifies the transmembrane potential Δ*V* of the internal cells (A vs. C and cell marked ‘2’). In this example, the well-separated cells (Figure 3A) shows a Δ*V* of 4.2 V, whereas the Δ*V* of the same cell in middle of the aggregated cells (Figure 3 C) is lower, i.e., 3.95 V instead of the external cells’ 4.65 V (Table 2). The same occurs for the 10 μm cells and a cell–cell distance of 1.2 μm (M case in Table 2). Moreover, in the aggregated cells, the stoma conductivity affects the Δ*V* that increases if *σ_S_* is higher (*σ_S_* from 0.2 to 1.3 S/m). This increment, observable from case C to case D, sees an increase of 0.7 V, whereas it is less evident in the well-separated cells, in which the Δ*V* increase is limited to 0.2 V (Δ*V* from 4.2 V to 4.4 V if the *σ_S_* varies from 0.2 to 1.3 S/m). Then, the effect of the stroma conductivity in case of the well-separated cells is limited. Results are reported in Table 2. In this table, cases with cell diameters equal to 10 μm and different cell distances, as well as different stroma conductivity, are also analyzed (cases from G to M). Focusing on the central cell, labeled ‘2’, and considering the 10 μm diameter, the transmembrane is not affected by large variations except for case M, the closest cells, for which Δ*V* is 0.914 V instead of 0.977 or 0.979 V in the cases of 100 and 1000 cell/mm^2^, respectively.

Figure 4 shows what happens to the transmembrane potential if a cell with a diameter of 50 μm is encapsulated in a medium with different electric conductivity, e.g., a fibrous shell, with respect to the other cells immersed in a myxoid medium. The represented cells have a local density of nine cells/mm^2^ with a cell–cell distance of 283 μm. In this case, the cell in the center of the nine-cell group is surrounded by a capsule of collagen, a fibrous stroma with a diameter of 200 μm. 

With respect to the homogeneous case, the potential electric distribution is deformed around the cell in the center of the nine-cell group. The transmembrane potential computed using Equation (3) is approximately 3.47 V, lower than the one numerically evaluated using the FEA simulation. In particular, the one obtained using FEA for the cell in the center of the group, the one labeled ‘2’ in Figure 4, is 4.4 V in the homogeneous case and 6.9 V for the cell surrounded by a fibrous shell, as the data in the table of Figure 4 report. Moreover, considering the cell labeled ‘3’ in Figure 4, which does not have a fibrous shell around itself, it can be noted that the transmembrane potential is close to that of the homogenous case (all of the medium around the cells has the same conductivity), 4.0 V vs. 4.4.V, respectively. Then, the FEA results suggest that the conductivity inhomogeneity affects the transmembrane potential. This numerical evidence in terms of electric field distribution was already discussed by the authors of [53]. In [53], the electric field distribution was computed and experimentally verified in a two-material sample (similar to the case in Figure 3E, where half of the model was potato and half was a gel with a different conductivity). The output was a modification of the electric field distribution at the border of the two materials, as can be seen in Figure 3E in the center close to the cell labeled ‘2’. Also, the transmembrane potential for cells immersed in media of different levels of conductivity is different, 7.3 V for the cell marked ‘1’ and 1.2 V for the cell marked ‘3’. 

### 3.2. Electroporation of Cell Cultures

**Figure 5:**
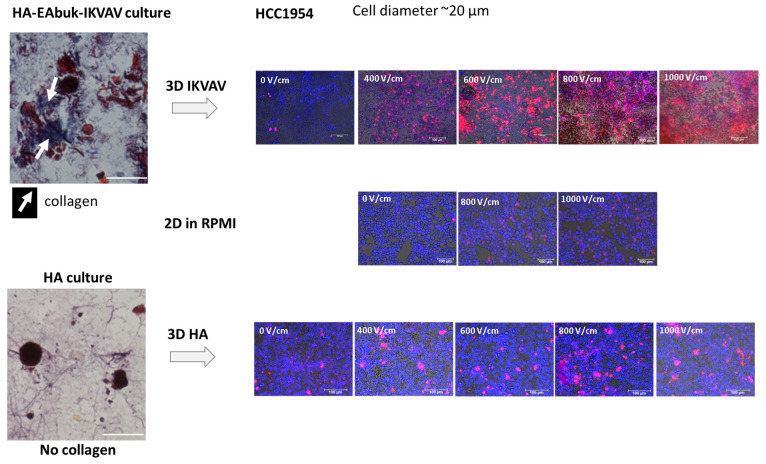
EP of HCC1954 cells cultured in HA-EAbuK-IKVAV- and HA-based hydrogels compared to 2D cultures. The **left panel** shows Masson trichrome staining, and the **right panel** shows PI and HOE staining after EP.

Figure 5 reports the experimental results obtained for the HCC1954 cultured in the HA or in HA-EAbuK-IKVAV scaffolds. The left panels show the Masson trichrome staining, performed 3 days after seeding, with the collagen fibers shown in blue. In particular, the cells cultured in the scaffold functionalized with EAbuK-IKVAV show collagen around the cells or around the cell spheroids. This abundance of collagen is not evident for the HA-only scaffold. In a previous author’s work [26], real-time PCR analysis showed an increment of Cola1a1 and LamB1 transcription only in the HA-EAbuK-IKVAV-based cultures as opposed to the HA-based and 2D cultures. Finally, the right panels of Figure 5 show the EP results at different electric field intensities from 0 to 1000 V/cm for, from top to bottom, the HA-EAbuK-IKVAV, 2D, and HA cultures, respectively. EP is carried out in presence of the culture medium RPMI with an electrical conductivity [26] close to 1.3 S/m. Red cells (PI staining) are permeabilized cells, whereas the only blue cells (Hoechst staining) are not permeabilized. In particular, the cells cultured in HA-EAbuK-IKVAV scaffolds in EP conditions uptake more PI and at lower electric field strength. In fact, the images in the first row of Figure 5 (cells cultured in HA-EAbuK-IKVAV scaffolds) show a larger number of red cells with respect to the cells cultured in 2D and in HA scaffolds. Therefore, the electroporation of the cell membrane is more effective in HA-EAbuK-IKVAV scaffolds with respect to the other cases. From Figure 5, it appears that the electroporation threshold for cells cultured in HA-EAbuK-IKVAV scaffolds is close to 400 V/cm (cells are stained in red in the panel related to 400 V/cm), whereas for cells in suspension or cultured in HA, the scaffold is higher than 1000 V/cm (the number of red cells increases in the last panel). Moreover, cells cultured in HA-EAbuK-IKVAV that show collagen production are permeabilized already between 400 and 600 V/cm, whereas the cells cultured in HA are not permeabilized at the same electric field intensity. As expected, control 2D cultures are not substantially permeabilized at any electric field intensity (no cells stained in red); in fact, only a few cells are red at 1000 V/cm. Then, the presence of collagen fiber, which has a conductivity close to 0.2 S/m [36], improves EP efficiency already at lower values of electric field intensity. In the literature, in order to electroporate cells in 2D adherent or suspension cultures, the cell medium is changed with an electroporation buffer (a solution of K_2_HPO_4_/KH_2_PO_4_, MgCl_2_ sucrose) [10,12] with a conductivity close to 0.2 S/m. We hypothesize that, in our cultures, the presence of the collagen fibers locally modifies the distribution of the electric field intensity since it modifies the local conductivity as already found through the simulation of real cases [27,39,54,55]. This fact is verified by culturing cells in HA-based scaffolds where collagen production is not experienced by the cells, as documented by the lack of blue areas in Masson trichrome staining and Cola1a1 in real-time PCR [26]. This situation is verified using FEA, considering both the cases of a single cell or a spheroid, respectively, surrounded by a collagen shell.

### 3.3. FEA vs. Experimental Results

**Figure 6:**
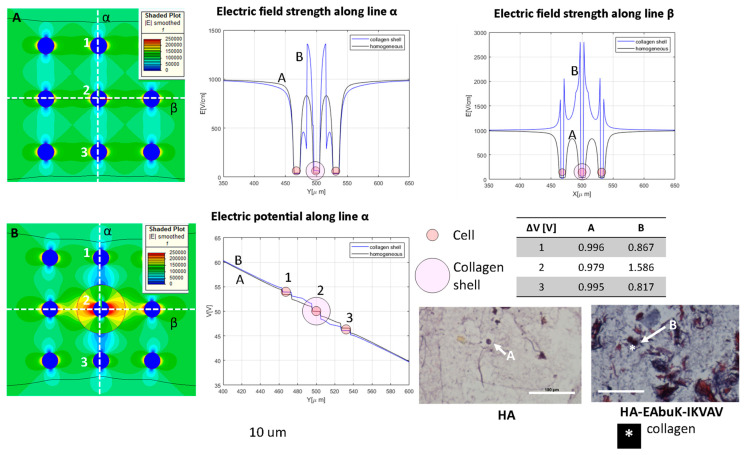
Color map of electric field intensity for the single-cell case with (**A**) no collagen shell, case H in Table 1, and (**B**) collagen shell, case around the central cell of the nine-cell group. Electric field and electrical potential sampled along line α and electric field along line β. Representative of the histological images (Masson trichrome staining) of the HA-only and HA-EAbuK-IKVAV HCC1954 cultures. Table reports transmembrane potential along line α. The number 1–3 represents the cells for which the transmembrane potential is evaluated.

**Figure 7:**
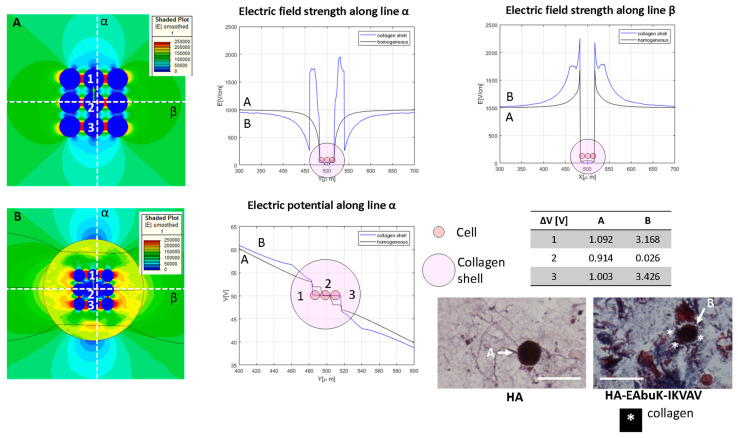
Color map of electric field intensity for the spheroid case with (**A**) no collagen shell, case M in Table 1, and (**B**) collagen shell around the nine-cell group. Electric field and electrical potential sampled along line α and electric field along line β. Representative of the histological images (Masson trichrome staining) of the HA-only and HA-EAbuK-IKVAV HCC1954 cultures. Table reports transmembrane potential along line α. The number 1–3 represents the cells for which the transmembrane potential is evaluated.

Figure 6 and Figure 7 analyze in detail the experimental case related to the HCC1954 culture in HA or in HA-EAbuK-IKVAV scaffolds using FEA. Figure 6 analyzes the case of a single cell eventually enclosed in a collagen shell, whereas Figure 7 analyzes the case of a spheroid with nine cells eventually surrounded by a collagen shell. The results obtained with the single cell or spheroid enclosed by a collagen shell are compared to the same results obtained with a homogeneous media around the cells, without any shell. In this FEA, the cell diameter was 10 μm, that is, the average diameter for the HCC1954 cells cultured in the scaffold (see authors’ previous work [26]). In the FEA, the cell density is 1000 cell/mm^2^ (cell–cell distance 21.6 μm) for the single-cell case and 8000 cell/mm^2^ (cell-cell distance 1.2 μm) for the nine-cell group, which simulates the spheroid.

In Figure 6, the electric field is represented in the color map, and it is sampled along the orthogonal lines α and β given the electric field strength behavior in the *y-* and *x*-axes shown at the top of Figure 6. Panel A represents the no-shell case, whereas in panel B the central cell has a collagen shell with diameter of 30 μm. The electric potential along the line α is evaluated for the homogeneous and collagen-shell cases. As evidenced by the graph of the electric field as a function of the spatial coordinate (generically indicated as X), the presence of the collagen shell, the blue line, increases the electric field strength close to the cell membrane with respect to the no-shell case. In fact, the color map B shows a higher value of the electric field around the central cell with respect the no-shell case (color map A). The same occurs for the electric potential along the line α. In fact, as reported in the table in Figure 6, the central cell has a transmembrane potential of 0.817 V and 1.586 V in the no-shell and shell case, respectively. The histological images (Masson trichrome staining) under the table in Figure 6 are representative of the single-cell case in the HA-only and HA-EAbuK-IKVAV cultures. The increase in the electric field intensity around the cell membrane due to the presence of electrical conductivity inhomogeneity, with a consequent increase in the transmembrane potential, can enhance the electroporation of the cell, as evidenced in Figure 5. In particular, the presence of collagen in the HA-EAbuK-IKVAV culture shows a lowering of the electric field threshold to obtain cell electroporation with respect to the culture on the HA-only scaffold. In fact, the cells cultured in HA only have the same round shape, diameter, and cell–cell proximity, also with the presence of some spheroids, as the cells cultured in the HA-EAbuK-IKVAV scaffold. The remarkable difference in these two cultures is the presence of collagen fibers only in the one produced on the HA-EAbuK-IKVAV scaffold. In this case, the effect on electroporation reported in Figure 5 is enhanced around the central cell labeled ‘2’.

The single cell surrounded by a collagen shell shows the role of the collagen shell in cell transmembrane potential. In Figure 7, the spheroids, in our case a group of nine cells close together, was analyzed. The spheroid is evaluated without (Panel A) and with (Panel B) a collagen shell with a diameter of 80 μm that encloses the entire cell group. The electric field map is shown in Figure 7, and it is sampled along the orthogonal lines α and β. The electric potential along the line α is also evaluated for homogeneous and collagen-shell cases. As evidenced by the graph of the electric field as a function of the spatial coordinates, the presence of the collagen shell, the blue line, increases the electric field strength close to the shell that encapsulates the cell groups with respect to the no-shell case. The color map B shows a higher value of the electric field around the group of cells inside the collagen capsule with respect the no-shell case (color map A). The same occurs for the electric potential along the line α. In fact, as reported in the table in Figure 7, the upper cell (labelled ‘1’) has a transmembrane potential of 1.092 V and 3.168 V in the no-shell and shell cases, respectively. Finally, considering both the cases with and without a collagen shell, the transmembrane potential of the central cell (labeled ‘2’) drops down with respect to that of the external cell labeled ‘1’. The histological images (Masson trichrome staining) under the table in Figure 7 are representative of the single-cell case in the HA-only and HA-EAbuK-IKVAV cultures. 

## 4. Conclusions

In this work, the simulation results highlight the effect of inhomogeneity on the transmembrane potential related to the application of an electric field in tissue. In particular, the presence of collagen around a cell increases the transmembrane potential, with respect to the homogeneous case. The same conditions are applied in the numerical model reproducing the presence of collagen around a group of cells. The experimental results show that EP could be improved by collagen produced by 3D cultures grown on HA-based hydrogels enriched with EAbuK-IKVAV. In fact, this is evident when comparing results of the HA-EAbuK-IKVAV scaffold with the ones determined in 2D adherent cultures or HA-based cultures lacking collagen, for which the transmembrane potential is not enhanced, and experimental results do not show electroporation at the same electrical conditions. Then, this work gathers the numerical analysis by FEA with experimental validation in a synthetic scaffold able to mimic ECM similar to the real tissues. This way, the presented method is able to show in an experimental platform the effect of the inhomogeneity of the tissue’s electrical properties during the application of the voltage pulses of ECT. 

## Figures and Tables

**Table 1:** Analysis set-up using FEA for different cases. Y = yes, the single cell or the cell group was studied both encapsulated in a collagen shell and without encapsulation; N = no, the collagen shell was not included. * model divided into two sub-areas with different conductivity; the number in the first row represents the conductivity of the shell and the one of cell interior.

	A	B	C	D	E *	F	G	H	I	L	M
***σ_S_* [S/m]**	0.2	0.2	0.2	1.3	0.2/1.3	0.13	0.2	1.3	0.2	1.3	1.3
***σ_C_* [S/m]**	0.13	0.02	0.13	0.13	0.13	0.13	0.13	0.13	0.13	0.13	0.13
**Cell diameter [μm], CD**	50	50	50	50	50	50	10	10	10	10	10
**Cell local density [cell/mm^2^], CLD**	9	9	350	350	9	9	1000	1000	100	100	8000
**Cell–cell distance [μm], CCD**	283	283	3.5	3.5	283	283	21.6	21.6	90	90	1.2
**Cell/cell-group shell**	Y	N	N	N	N	N	N	Y	N	N	Y

**Table 2:** Transmembrane potential, Δ*V*, in some relevant cases evaluated in the central cell-line (vertical) of the group of nine cells.

	A	B	C	D	E *	F	G	H	I	L	M
***σ*** **(*S*) [S/m]**	0.2	0.2	0.2	1.3	0.2/1.3	1.3	0.2	1.3	0.2	1.3	1.3
***σ*** **(*C*) [S/m]**	0.13	0.02	0.13	0.13	0.13	0.13	0.13	0.13	0.13	0.13	0.13
**CD [μm]**	50	50	50	50	50	50	10	10	10	10	10
**CLD [cell/mm** **^2^** **]**	9	9	350	350	9	9	1000	1000	100	100	8000
** Δ*V*** **(2) central**	4.2	2.9	3.95	4.1	7.3–1.2	4.4	0.968	0.979	0.968	0.977	0.914
** Δ*V*** **(1) external**	4.2	2.9	4.65	4.9	7.3	4.4	0.987	0.996	0.971	0.981	1.092
** Δ*V*** **(3) external**	4.2	2.9	4.65	4.9	1.2	4.4	0.987	0.995	0.971	0.981	1.003

* model divided into two sub-areas with different conductivity; the number in the first row represents the conductivity of the shell and the one of cell interior.

## Data Availability

Data are not available.
